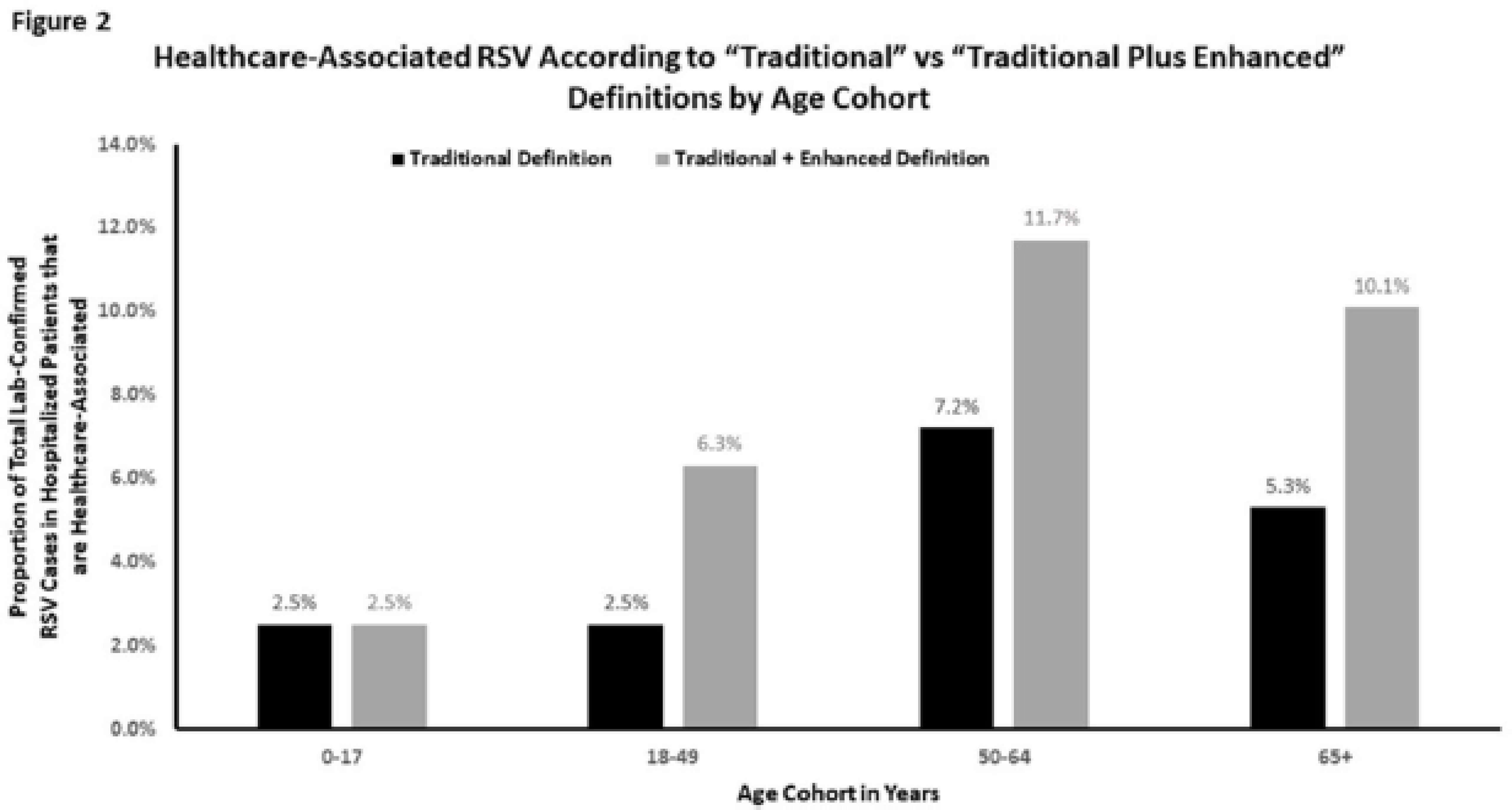# Respiratory Syncytial Virus: An Underrecognized Healthcare-Associated Infection

**DOI:** 10.1017/ash.2021.150

**Published:** 2021-07-29

**Authors:** Erin Gettler, Thomas Talbot, H. Keipp Talbot, Danielle Ndi, Edward Mitchel, Tiffanie Markus, Bryan Harris, William Schaffner

## Abstract

**Background:** Despite significant morbidity and mortality, estimates of the burden of healthcare-associated viral respiratory infections (HA-VRI) for noninfluenza infections are limited. Of the studies assessing the burden of respiratory syncytial virus (RSV), cases are typically classified as healthcare associated if a positive test result occurred after the first 3 days following admission, which may miss healthcare exposures prior to admission. Utilizing an expanded definition of healthcare-associated RSV, we assessed the estimates of disease prevalence. **Methods:** This study included laboratory-confirmed cases of RSV in adult and pediatric patients admitted to acute-care hospitals in a catchment area of 8 counties in Tennessee identified between October 1, 2016, and April 30, 2019. Surveillance information was abstracted from hospital and state laboratory databases, hospital infection control databases, reportable condition databases, and electronic health records as a part of the Influenza Hospitalization Surveillance Network by the Emerging Infections Program. Cases were defined as healthcare-associated RSV if laboratory confirmation of infection occurred (1) on or after hospital day 4 (ie, “traditional definition”) or (2) between hospital day 0 and 3 in patients transferred from a chronic care facility or with a recent discharge from another acute-care facility in the 7 days preceding the current index admission (ie, “enhanced definition”). The proportion of laboratory-confirmed RSV designated as HA-VRI using both the traditional definition as well as with the added enhanced definition were compared. **Results:** We identified 900 cases of RSV in hospitalized patients over the study period. Using the traditional definition for HA-VRI, only 41 (4.6%) were deemed healthcare associated. Adding the cases identified using the enhanced definition, an additional 12 cases (1.3%) were noted in patients transferred from a chronic care facility for the current acute-care admission and 17 cases (1.9%) were noted in patients with a prior acute-care admission in the preceding 7 days. Using our expanded definition, the total proportion of healthcare-associated RSV in this cohort was 69 (7.7%) of 900 compared to 13.1% of cases for influenza (Figure 1). Although the burden of HA-VRI due to RSV was less than that of influenza, when stratified by age, the rate increased to 11.7% for those aged 50–64 years and to 10.1% for those aged ≥65 years (Figure 2). **Conclusions:** RSV infections are often not included in estimates of HA-VRI, but the proportion of cases that are healthcare associated are substantial. Typical surveillance methods likely underestimate the burden of disease related to RSV, especially for those aged ≥50 years.

**Funding:** No

**Disclosures:** None

**Figure 1.**
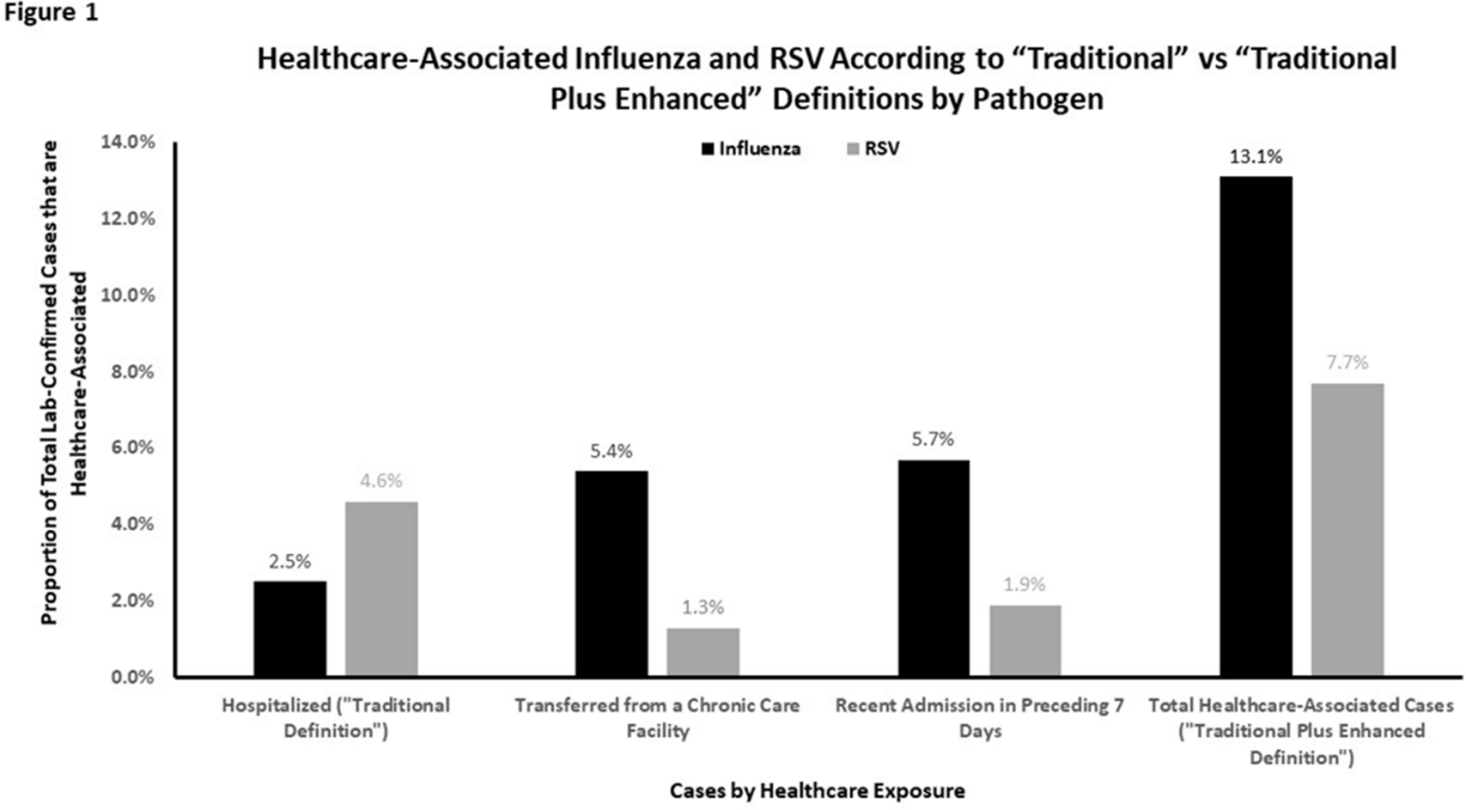

**Figure 2.**